# Direct to consumer testing in reproductive contexts – should health professionals be concerned?

**DOI:** 10.1186/s40504-014-0018-3

**Published:** 2015-04-29

**Authors:** Heather Skirton

**Affiliations:** Faculty of Health and Human Sciences, Plymouth University, Drake Circus, Plymouth, PL4 8AA United Kingdom

**Keywords:** Direct-to-consumer, Non-invasive prenatal test, Reproduction, Genetic test, Ethics

## Abstract

Direct to consumer genetic testing offered via the Internet has been available for over a decade. Initially most tests of this type were offered without the input of the consumer’s own health professional. Ethical and practical concerns have been a raised over the use of such tests: these include fulfilling the requirement for informed consent, utility of results for health care management and the potential burden placed upon health services by people who have taken tests.

These tests now have an application in reproductive healthcare. The advent of non-invasive prenatal testing has facilitated the genetic testing of the fetus using only a maternal blood sample. However, companies offering such tests, for example for aneuploidy, appear to be doing so based on a referral from the mother’s health professional. Preconception or prenatal carrier testing for a range of autosomal recessive conditions can be purchased without the input of a health professional who knows the prospective parents. However, unless the appropriate mutations for the specific population are included in the test, results may create false reassurance. Paternity testing without the consent of the putative father is also available via the Internet, as are tests to ascertain the sex of the fetus, which may be used to select children of a specific gender.

Direct-to-consumer tests may support prospective parents to identify genetic risk to their future children, however, it is important that they are aware of the possible limitations, as well as advantages, of these tests. National regulation may not prove effective in ensuring the safety of all individuals involved, therefore international pressure to ensure companies conform to Codes of Practice may be needed, especially in relation to tests that could influence reproductive decisions. However, health professionals have a duty to ensure they are sufficiently knowledgeable to enable them to guide patients appropriately.

## Introduction

For over a decade, it has been possible for individuals to purchase genetic and/or genomic tests over the Internet: these are termed direct-to-consumer (DTC) tests (Borry et al. 2010). In a commercial transaction, the consumer pays the company directly and it is possible to obtain tests without the involvement of a health professional (Eng and Sharp 2010), although increasingly companies are offering health professional contact before and or after the test is performed, and some now require a referral from the consumer’s doctor or health professional (Borry et al. 2010). Because of the shift in approach to include health professional input, the term ‘direct to consumer’ now describes a range of tests that are more directly accessible to consumers than those offered via the existing health services, with or without the involvement of a health professional. In the pharmacogenomics context, the term ‘beyond-the–clinic’ (BTC) has been suggested as an alternative to DTC (Prainsack and Vayena, 2013), however as this term is not yet generally employed, in this paper ‘DTC’ will be used.

Many DTC companies offer a panel of genetic or genomic tests that are purchased *en bloc* by the consumer, making consent procedures a challenge (Bunnik et al. 2012). The combination of different tests varies according to the company, but the panel frequently comprises tests for gene mutations for monogenic disorders, carrier tests for autosomal recessive conditions, tests for gene variants that could indicate susceptibility to a number of common diseases (Cornel et al. 2014), pharmacogenomic tests (Chua and Kennedy 2012) and sometimes tests that indicate specific physical characteristics, such as ear lobe thickness (Samuel et al. 2010). Of these, the tests that are of most relevance in the reproductive context are carrier tests that may identify the potential risk of any offspring having an autosomal recessive condition.

There have been significant ethical concerns raised over the marketing and use of DTC tests (Caulfield and McGuire 2012). Marketing is often based on enhancement of personal empowerment, with claims that use of the tests will enable individuals to have more control over their own health (Annas and Elias 2014). However, these claims have recently been brought into the spotlight with the warning to 23andme™ (23andme 2014) by the FDA that they could not substantiate the claims they made about the tests they offered (Annas and Elias 2014). It is possibly not surprising that three of the best known DTC companies, 23andme™, DeCODEme™ (deCODEme 2014) and Navigenics (Navigenics 2014) are not at present offering DTC testing for susceptibility to common diseases, but some continue to offer tests related to pregnancy, such as gender and paternity testing.

A series of three systematic reviews on DTC genetic and genomic testing in preparation for the development of a decision tool to support health professionals to advise patients considering a DTC test (Jackson et al. 2014): this is described later in the paper. When undertaking the review on service users’ views (Goldsmith et al. 2013), we found that there was a paucity of evidence on this topic and those studies that have been undertaken chiefly reported views of individuals with a professional interest in genetics or those who had been offered tests free or at a reduced price. In addition, many of the studies on user motivation involved potential, rather than actual, users of DTC tests. The available evidence on motivations for purchasing a DTC test indicated that users wish to obtain information about their own genetic makeup due to curiosity, to have more detail about risks for specific conditions (such as heart disease) or to help them manage their own health, for example through informing lifestyle choices. Interestingly, informing reproductive choice was not perceived as a motivating factor for use of DTC tests. While users were generally enthusiastic about the tests, there was little evidence that the results did help them to manage their health more effectively. However, it may be significant that in a study of 3640 individuals who were asked about their attitudes towards having a DTC personalised genetic risk assessment, those working for healthcare organisations were more concerned about potential results than others in the sample (Bloss et al. 2010).

In a review of health professional attitudes towards and experiences of DTC (Goldsmith et al. 2013), health professionals appeared wary about the use of DTC tests, although many of those studied had no experience of working with patients who had used them. Those working in the United States were more likely to support access to DTC than those outside that country. This may be related to perceptions of access to healthcare, as some health professionals felt that having direct access to genetic testing could reduce some of the inequities of health services, although it is difficult to see how those patients who do not have good access to clinical care could afford and/or benefit from DTC testing.

In a third review of existing policies on DTC testing, the published views of professional organisations and bioethics committees were synthesised (Skirton et al. 2012). The majority of organisations urged caution over the use of such tests, citing a lack of demonstrable benefit for consumers, risks of false reassurance or unwarranted anxiety and additional strain on the existing health services as negative outcomes of tests. Professional organisations and bioethics committees have highlighted the necessity of information (Table 1) and counselling to accompany DTC testing, emphasising that this should be available both pre and post-test (Skirton et al. 2012). However, this may not be perceived as important by consumers. In a study of consumer uptake of counselling services to accompany DTC testing, Darst et al. (2013) found that only 14.1% of their 1325 respondents had used the counselling services offered by the company. Many of those who did not utilise this option felt they already understood their results, however, the authors were unable to assess whether this was actually so and if DTC results were used as a basis for reproductive decisions , correct understanding of the results would be crucial.

While it may not be appropriate to align DTC tests with the same standards that would be required to obtain informed consent in a clinical context, some key issues should be understood by patients to reduce the chance of harm. The first is that although some mutations for single gene disorders may be included in the test, the company may not include all potential mutations (Cornel et al. 2014), or even those that are most relevant for the particular population to which the consumer belongs. This could result in prospective parents being falsely reassured that they do not carry a recessive condition that could affect their future children. The second point is that the tests for susceptibility to common disorders may be based on studies that indicate only weak associations between the genomic variant and the condition (Moonesinghe et al. 2011), or may include conditions with low heritability (Janssens and van Duijn 2010). Thus, the power of such tests to predict the likelihood of the individual developing one of the common diseases is often uncertain. These factors all indicate a need for regulation of DTC testing and this is consistent with the views of health professionals (Goldsmith et al. 2013). However consumers demonstrate a degree of ambiguity around regulation: Bollinger et al. (2013) discovered that while consumers wanted regulation and government oversight of DTC companies, they also wanted unrestrained access to DTC testing.

**Table 1:** Information required by consumers to make an informed decision about DTC testing (H. Skirton et al., 2012)

**Information related to genetic condition**	**Information related to test**	**Security issues**
General information about genetics	Purpose and nature of the test(s)	Confidentiality of results
Availability of treatment or lack of treatment	Risks associated with testing	Management and care of the sample
Implications of the result	Clinical utility of tests	Subsequent use or storage of sample
Possible impact on insurance or employment	Scientific evidence supporting use of tests	Quality regulation of the laboratory
	Availability of test-associated counselling by health professionals	
	Format and presentation of results	
	Sources of independent information	

### DTC in reproductive contexts

While DTC tests are offered and used in a range of contexts, some individuals or couples may wish to use them to support reproductive choice and decision making. Here I will discuss their potential use during the preconception and/or prenatal periods.

#### Prenatal testing using DTC and NIPT

The advent of fetal testing using cell-free fetal DNA in maternal serum has created feasible options for DTC prenatal testing (Hill et al. 2012a). Prior to the development of techniques to analyse fetal material in maternal circulation, invasive methods to extract fetal material for testing were required, necessitating the involvement of the mother’s personal health professional to take a sample (Skirton and Patch 2013). de Jong et al. (2010) raised alarms about the potential societal impact of the use of DTC tests in this application. However, the recommendations by European Academies Science Advisory Council (EASAC) and Federation of European Academies of Medicine (FEAM) (2012) specifically exclude the use of DTC for prenatal testing for health-related purposes, stating that the implications of the result and consequences of the test are so profound that it would not be appropriate to provide prenatal diagnosis without the input of relevant health professionals to provide quality care (Section 4.1.2, p17,18). Perhaps partly due to these recommendations, DTC companies offering tests such as Harmony™ (Ariosa 2014), Panorama™ (Panorama 2014), Verifi® (Illumina 2014) and MaterniT21™ (Sequenom 2014) do not at present appear to be offering NIPT in Europe as a direct-to-consumer test without a health professional referral. Thus, NIPT tests can be ordered, to detect aneuploidy such as Down syndrome in the fetus for example, but only via the mother’s health professional. However, the marketing emphasis is on reassurance for the parents, rather than the chance of detecting the condition. For example, the Panorama™ website (Panorama 2014) states ‘The Panorama prenatal test: because you deserve the reassurance that comes from having the most accurate and comprehensive genetic information available’. The onus is on the mother’s health professional to ensure she is aware that, beside the chance that the test result will reassure her, there is also the chance that the result may show the fetus is affected.

#### Pre-conceptual or prenatal carrier tests

One application of DTC testing that could be of genuine use to consumers is the option of carrier testing for autosomal recessive conditions such as cystic fibrosis, thalassaemia and spinal muscular atrophy. These conditions are individually rare and the chance of having a child with another carrier of the same condition is low, unless the parents are biologically related (Ten Kate et al. 2010). This means that unless an affected child is born into the family, carriers are generally unaware of their status. It is generally preferable that carrier testing to determine the genetic carrier status of the parents is undertaken prior to pregnancy to allow the couple more reproductive options and more time to make decisions, however, carrier testing is also possible during the pregnancy. This section relates to parental carrier testing in both scenarios.

In section 4.1.3 of the EASAC/FEAM recommendations (European Academies Science Advisory Council and Federation of European Academies of Medicine 2012) it is stated that genetic counselling is an essential component of preconception carrier testing to enable the individual to understand the risks to themselves and their families. The report further states that the ability of DTC companies to provide the requisite counselling is suspect, therefore it is highly preferable that it is carried out within the public health service sector and DTC companies should actually advise potential customers of this recommendation.

There are substantial differences in the products provided by companies offering carrier testing. At the time of writing, ‘Counsyl’ (Counsyl 2014), based in the Netherlands, offered tests for 100 conditions for $599 (US), while ‘Gentle’ (Gentle, 2014) provided testing for 1700 conditions for $1990. With regard to pre and post test counselling, information on the Counsyl website states that counselling is complimentary, but the nature of that counselling is not clear. Gentle uses a service called ‘Royal Doctors’ (Royal Doctors 2014) to provide telephone counselling to patients.

While carrier testing for a range of recessive conditions offers the chance to determine the genetic risk (if one exists) to future children, as previously stated, it is important that the test cover those mutations of relevance to the population (Cornel et al. 2014). The consumer therefore needs to ensure that the mutations relevant to his or her ethnicity are tested. In addition, any results that could be acted upon (for example if prenatal diagnosis is planned) should be obtained from an accredited laboratory using health service standards (European Academies Science Advisory Council and Federation of European Academies of Medicine 2012).

The reproductive health services offered in the consumer’s country could also be relevant to the consumer’s decision to purchase a DTC preconception or prenatal test. For example, prenatal diagnosis, preimplantation diagnosis and/or termination of pregnancy for fetal abnormality may or may not be available to the couple. The options for pregnancy management may influence their request for carrier testing and individuals who are made aware, for example, that termination of pregnancy would not be possible may decide against testing on the grounds that it may increase their anxiety without offering them a chance to influence the outcome of the pregnancy.

In choosing DTC carrier screening, consumers should also be informed that the panel of tests may include tests for mutations that cause adult-onset disorders, such as hereditary breast and ovarian cancer. They may therefore receive unwanted results of relevance to their own and their biological relatives’ future health.

#### Paternity testing and gender identification

While DTC companies are not currently offering prenatal testing for genetic conditions in Europe, a number are offering tests for paternity and identification of fetal sex, often called gender testing, using NIPT. In the United Kingdom, paternity testing is regulated under the Human Tissue Act (2004) and it is illegal to undertake it without the father’s informed consent, due to the many psychological, social and legal implications of the results. The use of maternal serum (containing maternal and fetal DNA) in combination with DNA obtained from the potential father would enable paternity testing to be undertaken during pregnancy without invasive testing and without the knowledge of the father.

These developments were predicted as early as 2009 in a report for the Public Health Genetics Foundation, where the authors (Wright and Burton 2009) alerted readers to the improbability of being able to ensure that paternity testing was only undertaken with the full consent of the putative father(s). In the same report, it was suggested that a Code of Conduct be established to protect individuals who might be involved in NIPT testing. However, a number of DTC companies are offering NIPT paternity testing and some openly advertise the option for the mother to maintain complete confidentiality and to send material from the father from which DNA can be extracted without his knowledge. In one example, the Prenatal Genetics Center (2014) state that for a cost of $990 they offer:

‘Full confidentiality of our testing: the mother can submit different samples from the alleged father(s) without asking him. Our laboratory is accepting hair samples, toothbrushes, semen stain and other forensic samples collected from the alleged father.’. Under the heading ‘Counseling’ on the same website, the company offers advice on the best types of paternal sample and optimal times to obtain samples, however the implications of the test results are not addressed.

The option of obtaining a paternity test result without the father’s consent could be seen to have some benefits for the mother, as she would be able to conceal any doubts about the paternity of her child that could disrupt her relationship with her partner. However, from the father’s perspective there could be both moral and legal objections, as having his genetic material tested without his knowledge could be seen as a violation of his human rights and could result in his alienation from a child he believes is his, without full explanation.

In other pregnancy related applications of DTC testing, some companies offer gender identification for the fetus, however feedback by consumers on the performance of some of them has been less than complimentary. For example, for one test offered by Gendermaker was marketing their test) via Amazon (Amazon.co.uk 2014a), but customer feedback indicated that many parents were unhappy with the reliability of the test (Amazon.co.uk 2014b) and what they felt was misrepresentation regarding the costs. There is a serious concern about the use of gender testing to identify and terminate a fetus that is not of the desired sex. In countries where it is illegal to perform an abortion based purely on the sex of the fetus, it is possible that parents could have a DTC test without the knowledge of a health professional and present with a request for abortion on other social grounds.

### So should health professionals be concerned about these developments?

It appears that the situation that would be most concerning from a counselling perspective, where prenatal tests for genetic conditions were offered without health professional input, is not currently a threat. Those companies offering NIPT for genetic conditions are doing so via a referral from the mother’s own health professional and this puts the onus for counselling for the test on that health professional, to comply with international recommendations (European Academies Science Advisory Council and Federation of European Academies of Medicine 2012). It also means that health professionals can exert consumer power if the tests are not of an acceptable standard and decline to use the company, but this does require them to make the appropriate investigations about suitability, specificity and sensitivity of each test before recommending the DTC company is used. They should also ensure that laboratories used by DTC companies are assessed as part of recognised quality assurance schemes (European Academies Science Advisory Council and Federation of European Academies of Medicine 2012).

Those companies offering tests for paternity or gender are still operating, but very susceptible to public review. The potential breach of law through offering paternity testing without the father’s consent is a problematic legal issue, but I would argue that it is not one that can or should be addressed via health services for two main reasons. First, paternity is not essentially a health issue and second, the nature of private paternity testing means it would be undertaken independently of health professional knowledge. Testing for fetal sex can be used to inform the status of a fetus at risk of a sex-linked disorder (Hill et al. 2012b), but this type of testing would be performed under the auspices of the health service. Testing for fetal sex purely for parental information is not the concern of health professionals, however the potential for termination of a fetus of a particular sex for social reasons raises ethical and legal concerns. Termination of pregnancy is a process that would involve health professionals and they therefore need to be aware of the existence of DTC gender testing outside the health service. This knowledge in itself should motivate health professionals to consider the possibility that prior knowledge of the fetal sex may motivate a request for termination and prompt them to explore the reasons for such a request.

Regarding use of DTC tests to assess carrier status, it is important for health professional and policy makers to be aware of the current situation with respect to DTC tests and to respond accordingly. This involves having the knowledge to guide or advise patients regarding the use of such tests. For this purpose, a decision tool (Skirton et al. 2014) was developed through consensus of an expert group to support health professionals in discussions with patients who might be considering a DTC test. It is suggested that health professionals who are faced with a patient asking for advice about taking a DTC test ask the patient ‘Why do you wish to have a DTC test?’. The decision tool was organised to provide information on the utility of using a DTC test in seven potential patient scenarios, based on the patient’s response to that question. With respect to reproductive issues, it is possible that prospective parents may be concerned about an existing genetic condition in the family or be members of an ethnic group with an increased risk of a specific disorder, in which case genetic testing through the health service would be advised rather than DTC testing. For those who are more generally concerned to reduce any risks to their potential offspring and wish to be tested for carrier status a wide range of autosomal recessive conditions, DTC testing may be a viable option. This tool is freely available on the EuroGentest website (Skirton et al. 2014) and can be accessed by both health professionals and by patients seeking information.

## Conclusion

Direct-to-consumer tests may support prospective parents to identify genetic risk to their future children, however, it is important that they are aware of the possible limitations, as well as advantages, of these tests. For tests offered via the Internet, national regulation may not prove effective in ensuring the safety of all individuals involved, therefore international pressure to ensure companies conform to Codes of Practice may be needed, especially in relation to tests that could influence reproductive decisions. However, health professionals have a duty to ensure they are sufficiently knowledgeable to enable them to guide or advise patients appropriately.

## Abbreviations

BTC: Beyond-the-clinic
DTC: Direct-to-consumer
NIPT: Non-invasive prenatal testing
EASAC: European Academies Science Advisory Council
FEAM: Federation of European Academies of Medicine
